# Characterization of infectious and non-infectious gastrointestinal disease in common variable immunodeficiency: analysis of 114 patient cohort

**DOI:** 10.3389/fimmu.2023.1209570

**Published:** 2023-08-30

**Authors:** David A. Sanchez, Karina Rotella, Crhistian Toribio, Matthew Hernandez, Charlotte Cunningham-Rundles

**Affiliations:** ^1^ Division of Allergy and Immunology, Mount Sinai, New York, NY, United States; ^2^ NYC Health + Hospitals/Metropolitan, New York, NY, United States

**Keywords:** CVID, gastrointestinal, noninfectious, infectious, enteropathy

## Abstract

Common Variable Immunodeficiency (CVID), a complex primary immunodeficiency syndrome defined by defective B cell responses to infection and vaccination, has heterogeneous clinical manifestations. Gastrointestinal (GI) complications in CVID, both infectious and non-infectious, can cause significant impairment leading to malabsorption and frank malnutrition. In order to better characterize the spectrum of GI disease associated with CVID, we describe 114 patients with GI disease (15.6%) from our 728 patient single center CVID cohort. Norovirus, Giardia and Cytomegalovirus were the most frequently isolated infectious pathogens. CVID enteropathy was the most encountered GI diagnosis based on endoscopy, with only a minority of patients having Crohn’s disease (6.1%) or ulcerative colitis/proctitis (4.5%). Concurrent autoimmunity (30.7%), lung disease (18.4%) and malignancy (8.7%) were also present in significant proportion of subjects. Lastly, 16 of 47 (34%) who underwent whole exome sequencing demonstrated a culprit gene defect associated with CVID.

## Introduction

Common Variable Immunodeficiency (CVID) is a heterogenous spectrum of genetic conditions leading to low or absent immunoglobulins, increased susceptibility to sinopulmonary infections, and for some, evidence of autoimmunity and increased risk of lymphoproliferative disorders. As a byproduct of B cell dysfunction, laboratory hallmarks of CVID include low IgG with concomitant low IgM and/or IgA, poor antibody responses following vaccination, markedly decreased isotype switched memory B cells (CD27^+^IgD^-^IgM^+^), and expansion of anergic B cells (CD21^Low^) (1). T cell defects have also been described in CVID including aberrant cytokine expression, low numbers of CD4 and CD8 T cells, and diminished cell proliferation (2, 3). Standard treatment entails administration of subcutaneous or intravenous immunoglobulin replacement therapy, which is generally efficacious and well tolerated. However, gastrointestinal (GI) manifestations of this immune defect are often debilitating, notoriously difficult to treat and contribute to heightened morbidity and mortality in patients with CVID.

Prevalence rates of GI complications in CVID have varied considerably. In a large study examining data on 2,212 patients from 28 medical centers contributing to the European Society for Immunodeficiencies Database, 77 of 901 patients (9%) were afflicted with enteropathy but the range for various centers was between 0 to 21% (4). However, gastrointestinal disease, both infectious and noninfectious in etiology, is one of the most universally cited complications in patients with CVID, and the range of GI pathology is vast. In our previous cohort of 473 patients with CVID studied over four decades, approximately 15.4% of patients had GI complications, including 5.9% with malabsorption, 4.2% with inflammatory bowel disease and 9.1% with liver disease such as hepatitis or hepatic granulomas (5). In this article, we describe the 114 patients who had GI disease from our current cohort of 728 CVID patients evaluated at the Mount Sinai Hospital, a quaternary-care academic center in New York City. We also discuss the recent literature on CVID related GI disease, including diagnosis and management of infectious and noninfectious sequela and the range of genetic defects implicated in CVID related GI disease.

## Methods

### Clinical and demographic information for CVID subjects

A retrospective chart analysis was conducted to characterize clinical and demographic information for 114 subjects with CVID associated GI disease. The data were collected *via* medical records as previously described from the current total cohort of 728 subjects (15.6%) (5). Subjects were evaluated at 1 of 2 centers, either the Immune Deficiency Clinic at The Mount Sinai Hospital or earlier at the Memorial Sloan-Kettering Cancer Center in New York City, New York. Demographic information including age, and sex were gathered from electronic medical records. The age of patients is given based on the year of birth. Subjects were confirmed to have CVID based on general consensus and previously published guidelines (1, 6). Confirmation of CVID required a significant decrease of IgG, of at least two standard deviations below the mean based on age, with concurrent decrease in either IgA and/or IgM for the vast majority of patients. Poor antibody responses to vaccination, initiation of symptoms after the age of 2, as well as exclusion of other identified causes of hypogammaglobulinemia supported the diagnosis of CVID (1). Disease onset is more difficult to define, but we used the first documented description of symptoms likely to be associated with CVID, such as recurrent sinopulmonary infections, or autoimmunity. The GI cohort described here includes only those patients where GI disease predominated as the most common complaint over time, based on chart review.

Immunoglobulin levels were collected prior to initiation of immunoglobulin replacement therapy in order to establish a baseline. All immunoglobulin levels that were below the quantitative assay’s lower limit of detection were converted to zero (i.e., an IgA level <5 mg/dL was changed to 0 mg/dL) for purposes of appropriate statistical analysis. Further immunologic characterization included phenotypic flow cytometry capturing numbers of total peripheral T cells, CD4+, and CD8+ T cells and IgM^−^IgD^−^CD27^+^ isotype switched memory B cells as a proportion of total peripheral B cells. GI disease was diagnosed by clinical history, stool studies, and endoscopy with mucosal biopsies as needed. All described subjects (n=114) had evidence of chronic Gl disease, both infectious and noninfectious, including chronic diarrhea, gastritis, inflammatory bowel disease (CVID enteropathy, Crohn’s disease or Ulcerative colitis/proctitis), malnutrition, and malabsorption, persisting for greater than 3 months. Frequently loose or liquid stools for 3 or more months with or without identification of an infectious agent qualified as chronic diarrhea. Malabsorption was defined as unintentional weight loss resulting in loss of more than 5 percent of typical body weight over 6 to 12 months, as well as laboratory evidence of nutritional deficiencies. Malnutrition was specified as malabsorption in addition to the requirement of total parenteral nutrition in an attempt to maintain weight above body mass index of 18.5. Liver disease was diagnosed *via* abnormal liver function tests, computerized tomography scans, sonographic imaging, specific PCR studies, and/or liver biopsy.

### Analysis

Descriptive data are highlighted as means, standard deviations, and associated ranges relative to reference values where applicable. Comparative studies assessing differences in immunologic parameters across certain demographic variables were conducted using nonparametric Mann-Whitney Wilcoxon tests calculated by Prism 9.5.1 software (GraphPad). Statistical significance was determined as traditionally defined, *P* < 0.05.

## Results

### Demographic data

The cohort of CVID patients afflicted with GI disease included a total of 114 subjects or 15.6% of the current total group; 82 subjects are alive, and 32 subjects are now deceased. There was a marginal predominance of female (n=60) versus male subjects (n=54), at 52.6% and 47.3%, a finding consistent with previously described non-GI specific adult CVID cohorts where females tend to be overrepresented, for reasons that remain unclear (7). The mean age for living subjects is 55 ± 16.5 years of age (range of 17–86 years). Notably, the average age of CVID disease onset for the entire cohort was 26 ± 16.7 years of age (range 3-42 years of age), which is harmonious with previously reported data for average age for initiation of symptoms related to CVID, both with and without associated GI disease (8). The age of disease onset for those living versus deceased was not significantly different (p=0.4408). Mean age of death for our cohort was 48 ± 18.2 years of age (range 11-82). As some patients were seen on consultative basis, long term follow-up for all is not available. While the causes of death for all subjects are not known, the majority were not specifically related to GI disease. Documented causes of death included progressive multifocal leukoencephalopathy following administration of high doses of steroids for treatment of recurrent immune thrombocytopenia (n=1), pulmonary hypertension in the setting of progressive inflammatory lung disease (n=1), spontaneous brain hemorrhage secondary to untreated lymphoma (n=1), Pneumocystis pneumonia in a patient with CD4+ count less than 200 (n=1), and undifferentiated shock (n=1).

### Infectious conditions in the GI CVID cohort

Both culture and/or PCR proven infectious agents involving the gastrointestinal tract were noted in this cohort (**Tables 1**). Chronic GI specific viral infections with Norovirus or Cytomegalovirus imposed some of the greatest burden of disease, with 8 patients (7%) and 3 patients (2.7%) affected, respectively. Other viral infections that co-occurred in subjects with GI disease were measles and shingles due to herpes zoster, (n=1 for each infectious process). Of note, parasitic infections included chronic giardiasis in 6 patients (5.2%), and less commonly, cryptosporidium in 1 patient (0.9%). Bacterial infections spanned a wide scope of pathogens and conditions including *Clostridium difficile colitis (n=1), Streptococcus bovis sepsis (n=1), Campylobacter* spp. *enterocolitis (n=1), Enteropathogenic E. coli (n=1), and Salmonella* spp. *infection (n=2).* Fungal infections were rare, with the exception of two life threatening opportunistic pathogens impacting non-GI organs*, Pneumocystis jiroveci* (n=2) and Cryptococcosis (n=1), leading to pneumonia and meningitis in those with GI predominant disease.

### Immunologic and genetic characterization

**Figure 1:**
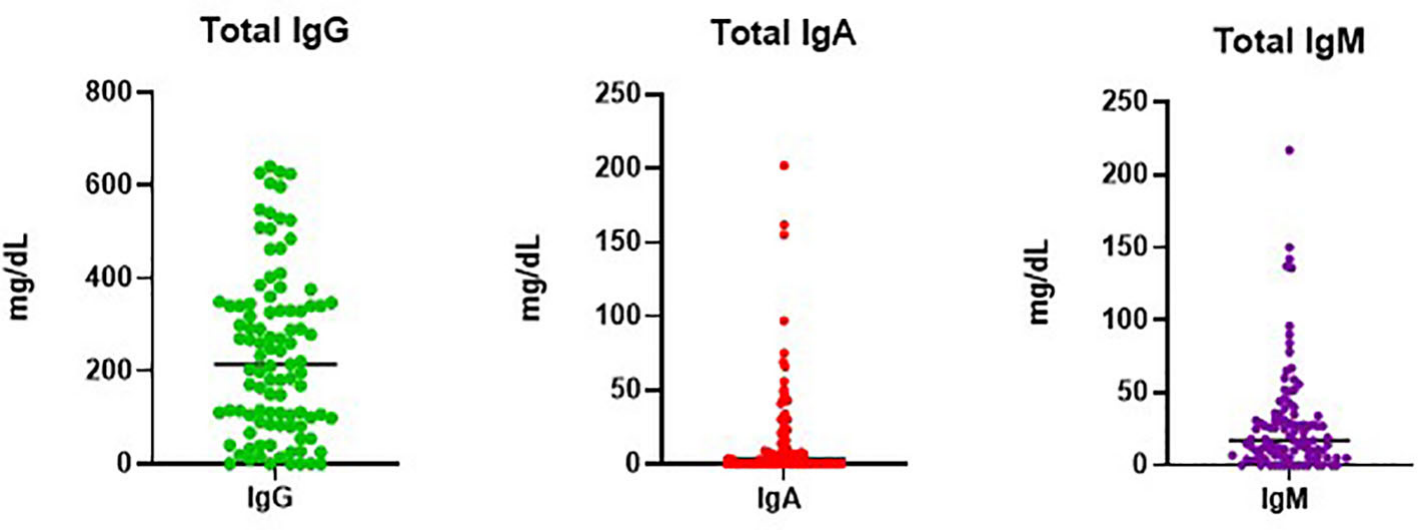
Immunoglobulin Levels in CVID with GI Disease.

Mean immunoglobulin levels for subjects showed similarly low mean IgG levels of 249 ± 170.6 mg/dL as noted in other studies (range 0-641 mg/dL, reference values 700 to 1,600 mg/dL) (**Figure 1**, **Tables 1**, **2**) (5, 9). Although the upper limit includes relatively higher levels of immunoglobulins, only 17 (14.9%) had IgG levels greater than 400 mg/dL. IgA and IgM levels were markedly decreased with IgM levels and IgA levels averaging to 22 mg/dL ± 26.8 mg/dL (range 0-150 mg/dL, reference values 70–400 mg/dL) and 13mg/dL ± 25.2 mg/dL (0-150 mg/dL, reference values 40–230 mg/dL), respectively (9). 90 (86.5%) patients had low IgA levels below the lowest limit of normal (40 mg/dL), including 49 (45.6%) with undetectable levels. Total B cell percentages for 79 subjects, averaged 10.2% ± 8.6% of total lymphocyte counts (range 0-37%, reference values 6%-19%), concordant with previously reported peripheral B cell counts measured in healthy subjects (**Table 2**) (10). As noted in this cohort, total peripheral B cell percentages may be normal in CVID, while B cell subpopulations such as switched memory B cells are more likely to be deficient, underscoring that these defects occur at later stages of B cell differentiation in CVID (11). Based on previous classification schemas that correlated percentage of switched memory B cells and certain clinical phenomenon associated with CVID such as autoimmunity, splenomegaly, lymphadenopathy and granulomas, B cell populations were reported as total, or populations containing less than 2% or 0.4% isotype switched memory B cells (10, 12, 13). Switched memory B cell percentages were determined for 62 subjects, showing an average of 3.28% ± 6.07%. Of these, 70.9% had less than 2% total (range 0-1.7%). Within the group of subjects with less than 2% switched memory B cells, 24 out of 44 (54.5%) had profoundly low levels of switched memory B cell percentages lower than 0.4%. Total peripheral T cell percentages varied substantially (n=79), with an average of 73% ± 13% (range 39-95%, reference values 61 to 85%) (14). CD4+ counts obtained from 20 patients within the cohort demonstrated a mean of 542.3 ± 301.2 cells/μl (range 96-1,154 cells/μl, reference values 500-1,500 cells/μl). Although CD4+ T cell lymphopenia has repeatedly been associated with gastrointestinal manifestations in CVID, this cohort only had three out of 20 subjects with less than 200 CD4+ cells/μl (15–17).

**Table 1:** Infectious pathogens isolated.

	No.	% of cohort (*n* = 114)
*Norovirus*	8	7.0
*Giardia* spp.	6	5.2
*Cytomegalovirus*	3	2.6
*Salmonella* spp.	2	1.7
*Campylobacter*	1	0.9
*Clostridium difficile*	1	0.9
*Cryptosporidium*	1	0.9
*Enteropathogenic E. coli*	1	0.9
*Streptococcus bovis*	1	0.9

**Table 2:** Immunologic parameters.

	Normal range	Median (range)
IgG (mg/dL)	700–1,600	249 (0−641)
IgA (mg/dL)	70–400	13 (0-162)
IgM (mg/dL)	40–230	22 (0−217)
T-cell populations		
CD3+, % (*n* = 79)	55–89	73 (39–95)
CD3+CD4+, cells/mm3 (*n* = 20)	480–1,700	542.3 (96–1,154)
B-cell populations		
CD19+, % (*n* = 79)	5–15	10.2 (0–37)
CD19+CD27+IgD–, % (*n* = 62)	6.5–29.2	3.28 (0–29)**

**44/62 had <2% Isotype switched memory B cells.

As whole exome sequencing became available, 47 subjects within this group underwent genetic analysis (**Table 3**). Of these, 16 had mutations associated with CVID (34%), including *NFKB1* (n=5), *TACI* (n=4), *NFKB2+TACI* (n=1), *IKZF1* (n=1), *ADA2* (n=1), *CTLA4 + IRFBP2* (n=1), *RAG1+RAG2* (n=1), *LRBA* (n=1) and *BACH2* (n=1). The mean age of disease onset for those with genetic defects was 35 years of age. Three subjects, all with *NFKB1* mutations, have died since collection of data. Average age of death for these 3 subjects was 67 years of age. One of these 3 patients died from undifferentiated shock and hypoxic respiratory failure without retrieval of an infectious agent, while the other two patients died from chronic debilitation. Mean immunoglobulins levels reflect the rest of the GI cohort, with the exception of IgA nearing undetectable levels (mean IgG 207 mg/dl, IgA 3mg/dl, IgM 22mg/dl). Switched memory B cells were also found to less than 2% for all 17 patients with identified gene defects, and the average CD4+ count was 383.6 cells/uL. 15 out of 16 had GI disease consistent with CVID enteropathy based on endoscopic findings, with only 1 patient having Crohn’s disease. 5 of 16 patients had a history of at least one episode of immune thrombocytopenic (ITP), and 3 of 16 had a history of Granulomatous and Lymphocytic Interstitial Lung Diseases (GLILD).

**Table 3:** Identified gene defects in GI CVID and associated clinical characteristics.

Gene Defect	Mutation	Age	Age of Death	Age of Symptom Onset	Sex	Associated Conditions
*IKZF1*	p.Ser385*	37		3	M	CVID enteropathy, previous history of T cell leukemia/lymphoma at age 3
*ADA2*	p.Gly47Arg	74		48	M	CVID enteropathy complicated by TPN dependence and chronic norovirus, protein losing enteropathy, cholelithiasis s/p cholecystectomy, splenomegaly, and pancytopenia
*RAG1, RAG2*	*RAG1*: p.Asn968Lys *RAG2*: p.Met110Leu	59		40	F	CVID enteropathy complicated by chronic norovirus, previous diagnosis of celiac’s disease, ITP complicated by splenectomy
*CTLA4, IRFBP2*	*CTLA4*: c.109 + 1G>A (splice donor), *IRFBP2*: p.Gln97His	60		45	F	CVID enteropathy
*NFKB1*	c.1301-1G>A (essential splicing)		51	42	M	CVID enteropathy, lymphopenia, neutropenia, aphthous ulcers, frequent pneumonias, lymphoid hyperplasia, and splenectomy
*TACI*	p.Leu171Arg	57		44	M	CVID enteropathy, GLILD, recurrent skin infections; pneumonias, sinusitis, and splenomegaly
*BACH2*	p.Arg666Lys	56		40	F	CVID enteropathy complicated by TPN dependence, chronic norovirus, and sinusitis
*TACI*	p. Ala181Glu	61		40	F	CVID enteropathy previously diagnosed as IBS, seizure disorder
*NFKB1*	p.Arg579Lys		80	64	F	CVID enteropathy, recurrent ITP complicated by splenectomy, lung granulomas and history of Strep Bovis sepsis
*TACI*	p.Leu69fs	46			M	CVID enteropathy, chronic fatigue and joint pain
*NFKB1*	c.928-2A>G (splice acceptor)	35		16	M	CVID enteropathy, recurrent ITP, chronic cough, and sinusitis
*NFKB1*	p.Thr869fs		72	60	F	CVID enteropathy complicated by TPN dependence, recurrent ITP complicated by splenectomy and AIHA, frequent giardia infections, and GLILD
*NFKB1*	(indel-frameshift)	32		11	F	CVID enteropathy, chronic Clostridium difficile, pneumonias, otitis media, herpes zoster at age 12
*TACI*	p.Cys104Arg	62		20	M	CVID enteropathy, ITP, splenomegaly
*NFKB2, TACI*	*NFKB2*: p.Gly719Glu *TACI*: c.204dup p.Leu69Thrfs*12	45			M	Inflammatory bowel disease (Crohn’s disease), gastric lymphoma and lung nodules
*LRBA*	p.Ala2784Gly/p.Met467Val	13		10	M	CVID enteropathy, GLILD, splenomegaly, uveitis, and liver granulomas

### Noninfectious conditions

**Figure 2:**
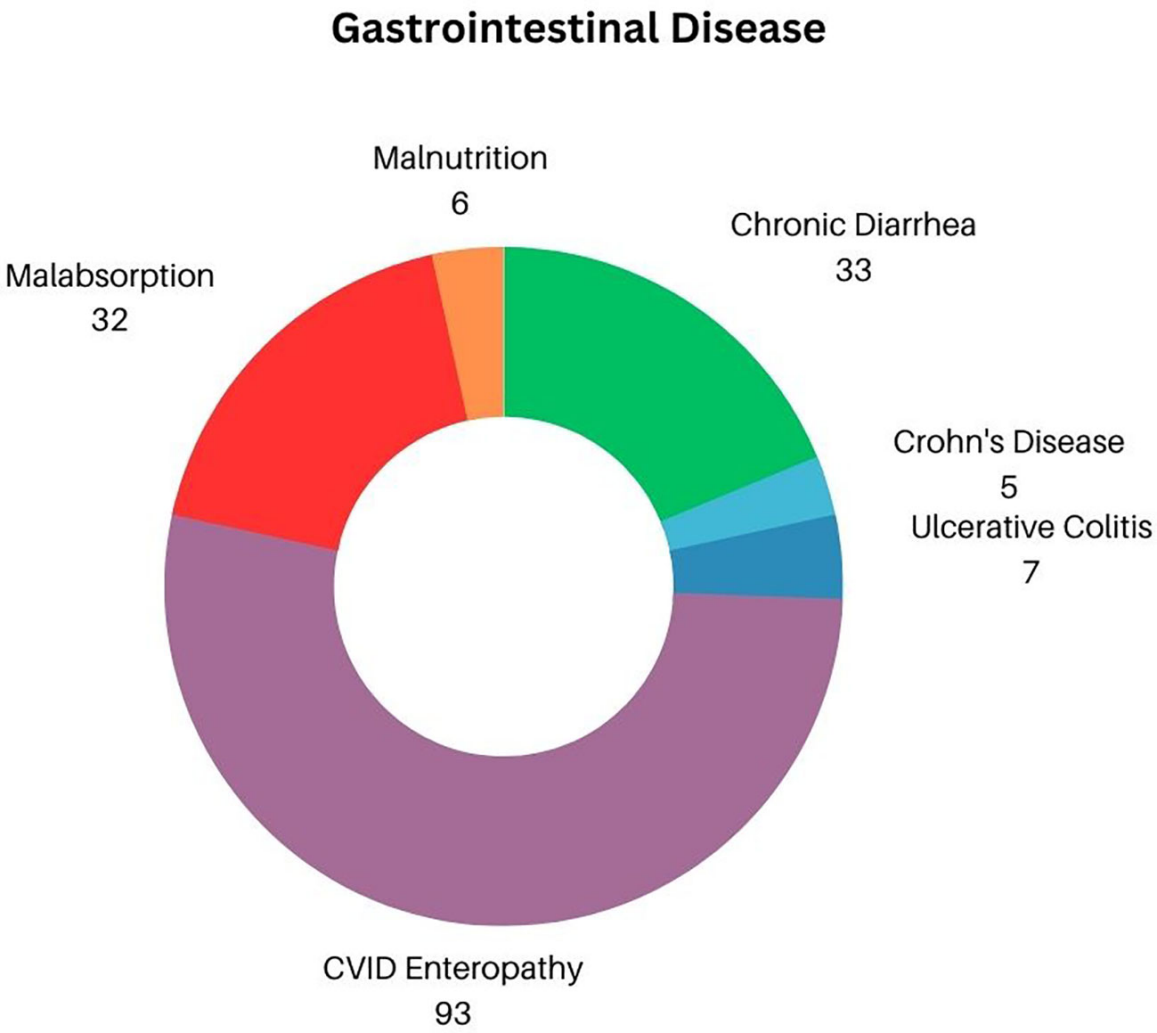
Distribution of GI Disease in CVID.

Chronic diarrhea (both infectious and noninfectious in etiology) was the main GI complaint, affecting 33 of 114 subjects (**Figure 2**) (28.9%). Inflammatory bowel disease, diagnosed by biopsy included ulcerative colitis/proctitis identified in 7 patients (6.1%) and 5 with Crohn’s disease (4.5%). Thus, 93 other patients (83%) were diagnosed with CVID enteropathy, or small bowel inflammation that does not fit the criterion used to diagnose either Crohn’s disease or ulcerative colitis/proctitis, but was defined by increased numbers of intraepithelial cells, absence of plasma cells, and/or villous atrophy (4, 17). As a likely secondary effect of either chronic diarrhea and/or inflammatory bowel disease, 33 subjects (28.9%) had evidence of malabsorption. For those most severely impacted, this progressed to frank malnutrition, defined as the need for total parenteral nutrition in 6 subjects (5.2% of total cohort, or 18.1% of those with evidence of malabsorption). Perplexingly, although chronic gastritis has previously been reported as mirroring the general population in patients with CVID, there were only 2 recorded cases (1.8%) of gastritis linked to autoimmune mediated pernicious anemia within our cohort (18, 19). Splenomegaly is often noted in CVID subjects with cytopenias—in fact, recent studies have demonstrated that up to 88% of cytopenias present simultaneously with splenomegaly (20). In this GI cohort, splenomegaly was specifically noted in 10 patients (8.7%).

Coexisting liver pathology in this GI cohort was also examined, highlighting conditions found in previous studies assessing liver disease in CVID (21, 22). In this cohort, ~17% of subjects demonstrated evidence of significant liver disease (n=19). On a more granular level, hepatitis was identified in 7 of 112 subjects (6.1%), with hepatitis C (PCR confirmed) in 2 subjects (1.7%). Nodular regenerative hyperplasia was identified in 4 patients on liver biopsy in this selected GI cohort (3.5%). Portal hypertension (n=2, 1.7%) progressed to end stage liver cirrhosis in only 1 person (0.9%) Liver granulomas were found on biopsy in 4 individuals (3.5%) while primary biliary cirrhosis was established in 1 patient (0.9%).

### Concurrent lung disease, autoimmunity, and malignancy

**Figure 3:**
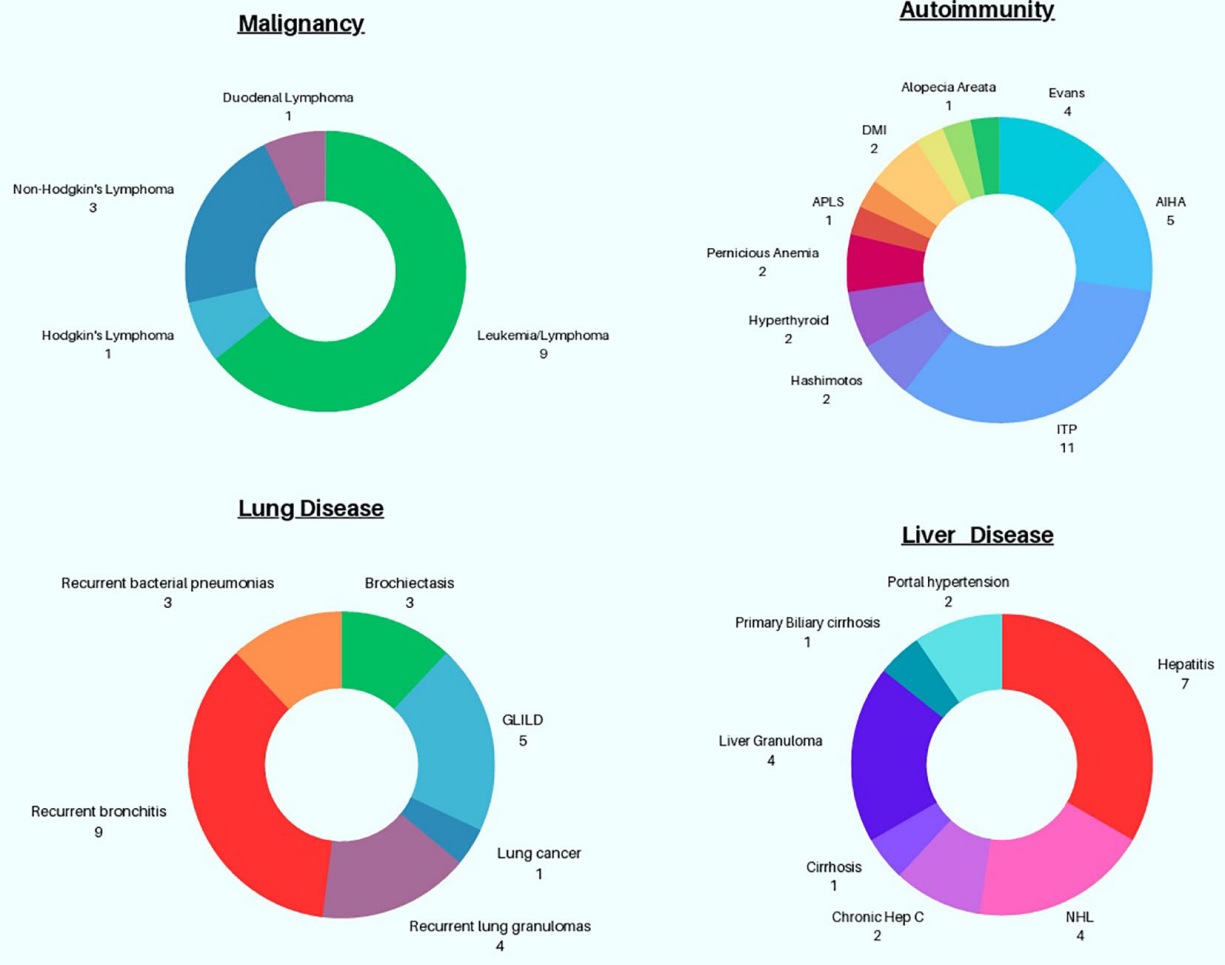
Graphical Representation of Non-GI associated Complications in patients with CVID GI disease.

Chronic lung disease often presents devastating consequences in CVID, contributing to increased morbidity and mortality, which can potentially be magnified in the setting of simultaneous GI disease (**Figure 3**) (5, 23). In this cohort, chronic lung disease was co-diagnosed with GI disease in 18.4% of subjects (21 out of 114). GLILD was documented in 5 patients (4.3%) with comorbid GI disease (23). Three of the 5 subjects diagnosed with GLILD were also identified as having lymphocytic interstitial pneumonia (LIP) subtype, which is one of the more rare granulomatous and lymphoproliferative histopathological patterns in GLILD that may portend a poor prognosis (24). Bronchiectasis, a major lung complication in CVID, was observed in 3 patients (2.6%), based on information from chart retrieval which may underestimate the frequency of this finding within our cohort.

Autoimmunity is a well described occurrence in CVID and was also noted in this cohort (25). 30.7% (n=35) subjects exhibited autoimmunity, superseding previously reported percentages of autoimmunity in the general CVID population across the United States (8, 25, 26). Cytopenias were commonest, with ITP observed in 11 subjects (9.6%) and autoimmune hemolytic anemia (AIHA) in 5 subjects (4.3%). Evan’s syndrome, or coexisting ITP and AIHA, was seen in 4 persons (3.5%). Intriguingly, 4 subjects (3.5%) also had been given the diagnosis of sprue (celiac disease). Other miscellaneous autoimmune findings observed in this cohort include hyperthyroidism (n=2), Hashimoto’s thyroiditis (n=2), antiphospholipid syndrome (n=1), diabetes mellitus type 1 (n=1), uveitis (n=1), morphea (n=1), presence of anti-neutrophil antibodies leading to chronic neutropenia (n=1), alopecia areata (n=1), and chronic aphthous ulcers (n=1).

Malignancy occurs in CVID, with a 5-to-12-fold increased risk relative to the general population, based on a recently published systematic review (27). Gastric cancers, non-Hodgkin’s lymphomas, and non-melanoma skin cancers have been cited as the most frequently encountered malignancies in CVID (27). Within our cohort, 10 distinct cancers were identified with leukemia/lymphoma prevailing as the most frequent; 9 patients had this diagnosis (7.8%), including 3 patients (2.6%) with non-Hodgkin’s lymphoma and 1 with Hodgkin’s lymphoma (0.9%). Surprisingly, although gastric cancers are rather prevalent within CVID, none were identified in this selected cohort, while 1 case of duodenal lymphoma was known. Finally, one patient in this cohort had lung cancer.

## Discussion

### Infectious complications

#### Norovirus

As alluded to previously, recurrent sinopulmonary infections are a hallmark of CVID. However, other devastating chronic infectious processes may predominate in CVID patients within the gastrointestinal tract. As illustrated in **Table 1**, chronic Norovirus, a positive single-stranded RNA virus and leading cause of acute gastroenteritis across the world, was identified in 8 subjects, or 5.4% in our cohort. Consistent with previously published case reports and our own clinical experience, eradication of Norovirus infections in these patients has proven to be difficult due to the lack of effective therapy (28). In another cohort of 8 patients with confirmed CVID enteropathy, unrelenting norovirus excretion was documented for more than 3 years, with only three patients’ demonstrating evidence of full viral eradication and reconstitution of normal villi on endoscopy (29). Of those three patients, 2 patients had received the antiviral nucleoside analogue ribavirin and one patient had spontaneous viral resolution. Anecdotally, ribavirin’s utility is curbed by ribavirin associated anemia, which occurs in 10% of all treated patients (i.e., including those that are receiving ribavirin for treatment of hepatitis C) and caused anemia in 4 out of 7 immunocompromised patients (30, 31). In a cohort of 11 immunocompromised patients, including 9 patients with CVID, lactose free diet, gluten free diet, high dose IVIG, nitazoxanide and ribavirin were tested (31). Overall, while there were varied responses, the majority of patients demonstrated no clear improvement. The antiparasitic nitazoxanide led to no meaningful effect in 6 out of 10 patients, relapse or increase in viral load in 2 out of 10 patients, and clinical improvement with viral clearance in only 2 out of 10 patients (31). Randomized clinical trials assessing efficacy of proposed treatments and further exploration of immunologic factors contributing to chronic norovirus within the CVID population will prove indispensable to ensure better outcomes.

#### Giardia spp.

Giardiasis, caused by the water-borne protozoan *Giardia lamblia*, is one of the most commonly cited gastrointestinal infections detected in CVID, in particular those with low levels of IgA (32). In our patient cohort, IgA levels were markedly diminished across a vast majority of the patients with greater than 85% of patients demonstrating levels below the lower limit of 40mg/dL. Undetectable levels of IgA were identified in nearly half of the patient cohort (45.6%), which included all patients who had a previously documented history of giardiasis infection. Intriguingly, beyond absence of IgA, the lack of IL-10 producing regulatory B cells in CVID may play a role in susceptibility to *Giardia*—recently, investigators developed an IL-10 deficient mouse model that showed intractable diarrhea, lethargy and weight loss following infection with *Giardia muris* in mice lacking IL-10 while wild type mice did not showcase any evidence of infection (33, 34). For those patients afflicted with Giardiasis in our cohort, treatment with metronidazole was generally adequate for elimination; nonetheless, 2 patients had recurrent episodes of giardiasis, with 1 patient developing persistent giardiasis for 7 years which was refractory to several courses of antibiotics. One of the largest studies detailing infections across several organ systems in CVID recorded 35 *Giardia* spp. infections out of a total of 252 infections (14%) over 4 years (35). Giardia infections comprised more than half of the total GI specific organisms identified (i.e., 35 out of 67 GI infections) (35). Reasons for the discrepancy between the rate of Giardia infection in our cohort and this study are unclear since both cohorts are from nations where Giardia prevalence rates are similar. A recently published case series of 4 patients and review of 17 previously published CVID Giardia infection case reports highlighted an incidence of ~12.9% in their cohort, all with undetectable or very low IgA (32). In a review of previously published case reports, 6 of 17 cases were refractory to metronidazole, requiring treatment with one or several of the following agents: ornidazole, albendazole, nitazoxanide, and trimethoprim/sulfamethoxazole (32). Alarmingly, treatment failure with nitroimidazoles, such as metronidazole or tinidazole, is on the rise with recent reports indicating up to 45% resistance depending on the geographical region (36). A recent systematic review appraising the most recently published literature regarding efficacious treatment of nitroimidazole resistant giardiasis included 663 patients, including 14 patients with HIV and 17 patients with IgA deficiency. Although most of the studies lacked randomization with comparator arms and were likely of low-quality evidence, the study highlighted how there have been documented successes of 67-78% clearance rates with combination anthelmintic benzimidazole (i.e., albendazole or mebendazole) and nitroimidazoles. Quinacrine as monotherapy also is a viable option as cure rates of 88% were achieved in the systematic review (36).

#### Cytomegalovirus

Cytomegalovirus (CMV), a herpesvirus leading to seropositivity in 40-100% of the world’s population, was the third most commonly identified pathogen leading to chronic GI infection in our cohort (37). Unsurprisingly, two of three patients had CD4+ counts less than 200 cells/μl and these patients also had a history of other opportunistic infections such as Pneumocystis pneumonia, leading to the death of one patient. A recent case series describing 34 patients with CMV associated complications in CVID drew attention to CMV’s proclivity for GI disease in CVID, with 25 of 34 CMV infections (73.5%) leading to enterocolitis (38). This finding parallels the type of CMV related pathology seen in the setting of hematopoietic stem cell or solid organ transplantation (38). In another smaller case series, 5 out of 6 CVID patients were diagnosed with CMV enterocolitis, with 4 succumbing to their illness (39). It is worth noting that all CVID patients in this cohort diagnosed with CMV had undergone iatrogenic immunosuppression for treatment of CVID associated autoimmune conditions like autoimmune cytopenias, highlighting the heightened risk of mortality posed by this pathogen in the CVID population (39). Based on this case series, 66.7% of subjects had monogenic CVID, with *NFKB* defects most commonly represented. Monitoring of disease is problematic as antibody levels cannot be assessed in patients on immunoglobulin replacement therapy and low or absent peripheral viral loads may be seen in active CMV infection among those receiving immunosuppression. Of great concern is the fact that ganciclovir and valganciclovir treatment complications include cytopenias, for which CVID patients are already at high risk; this treatment side effect poses a major limitation on length of treatment (for instance, lifelong suppressive therapy for CMV if necessary) and requires careful laboratory monitoring upon initiation of this first line therapy. Additionally, CVID associated enteropathy may lead to poor absorption of oral valganciclovir and has the potential of precipitating further drug resistance making it imperative to consider intravenous medications for treatment of CMV in the setting of CVID associated GI disease (38). Similarly, use of foscarnet is limited by risk of anemia and nephrotoxicity, and other agents such as maribavir, leflunomide, brincidofovir, letermovir and Artesunate have limited efficacy or are cost prohibitive (40).

#### Other infections

*Salmonella, Campylobacter, Clostridium difficile, Cryptosporidium, Enteropathogenic E. coli* and *Streptococcus bovis* were other pathogens identified in this GI specific CVID cohort. It is worth noting that the literature lists *Salmonella*, and *Campylobacter* as contributing significantly to the burden of GI infections in CVID (41, 42). These infections are likely to cause diarrhea in others in our CVID patient population, but laboratory testing-based pathogen identification may have not occurred due to lack of testing or appropriate clearance of both of these pathogens. Furthermore, this cohort is focused on those patients with chronic GI conditions, rather than transient GI infections.

### Noninfectious complications

#### CVID enteropathy

The majority of patients with chronic GI disease in this cohort were labeled as having CVID enteropathy (n=93, 81.5%), a loose diagnostic term with no clear criteria. However, the usual findings on endoscopic biopsy include lymphoid and/or neutrophil infiltrates, reduced or absent intestinal plasma cells, GVHD-like crypt apoptosis, and villous blunting, the features noted in our CVID patients (43). Often, CVID enteropathy develops concomitantly with chronic infections, begging the question whether infectious agents have the ability to precipitate or prolong CVID enteropathy. Previous studies have shed light on other potential biomarkers that may have utility in assessing proclivity or severity of GI disease in CVID (44). In particular, there is immense interest in identifying markers of host-commensal disruption. Recently, our group provided evidence of increased zonulin and intestinal fatty acid binding protein (I-FABP), two proteins implicated in gut barrier dysfunction, in 33 CVID patients with and without CVID enteropathy (45). In the context of likely gut barrier dysfunction, circulating bacterial DNA was also identified across the cohort; ultimately, there was no difference in the levels of bacterial DNA or gut barrier associated proteins based on the presence or absence of enteropathy (45). This finding lends credence to the idea that gut barrier compromise may be consistently found across most patients with CVID leading to host-commensal imbalance, regardless of GI specific clinical symptoms. Nonetheless, studies examining the presence of these biomarkers in larger cohorts would help to further validate this finding.

In our patient cohort, 3.5% patients (n=4) had been diagnosed with sprue based on clinical history such as intolerance to wheat, rather than by endoscopic analysis. Of interest, histo-molecular features reminiscent of sprue are encountered in CVID enteropathy, including increased TCRγδ+ intraepithelial lymphocytes (IELs) in the descending duodenum with villous atrophy (18, 46). Beyond clinical history of wheat intolerance, HLA typing and gene-expression microarray analyses of intraepithelial lymphocytes may provide more objective evidence to facilitate separation of these two disease entities in CVID (47). Nevertheless, although certain markers can provide evidence for the diagnosis of sprue in CVID, it remains controversial whether patients with CVID enteropathy do develop sprue. For instance, while finding HLA DQ2.5/DQ8 typing could support a sprue diagnosis, serological testing for sprue-specific antibodies cannot accurately diagnose the illness in CVID due to antibody defects (48). Determining responses to a gluten-free diet as a diagnostic tool in CVID have also been controversial, as findings have been inconsistent (48).

Treatment of CVID enteropathy is challenging. Current management includes topical budesonide, mesalamine, and antibiotics targeting small intestine bacterial overgrowth, though these therapies lead to highly variable results (49, 50). While biologics are commonly used in IBD in general, randomized clinical trials demonstrating their efficacy in CVID are lacking, though improvement has been reported anecdotally in case reports. There has also been reluctance to use some biologics (i.e., TNF inhibitors) due to the potential risk of opportunistic infections (51). While vedolizumab, a gut specific IgG1 monoclonal antibody to α4β7 integrin poses little opportunistic infection risk, resulted in clinical improvement and endoscopic remission in 2 of 3 patients with CVID enteropathy in 2017 (49), on a follow up study in 7 of our patients in 2020, only one patient demonstrated sustained clinical benefit, 3 patients experiencing acute deterioration after initiating therapy and 3 others discontinuing after lack of improvement (46). Anecdotally, abatacept has been used with benefit in our one patient with compound heterozygous mutations in LRBA. However, ordering abatacept for treatment of CTLA-4 or LRBA associated GI disease remains difficult in the United States as the medication is not approved for this indication. Finally, compelling evidence links chronic treatment with antibiotics, pervasive use of anti-inflammatories, and a dysregulated immune microenvironment to increased likelihood of a perturbed gut microbiome in CVID (49). Therapeutic approaches are still being examined to modify the composition of the gut microbiome which may prove to be beneficial, such as high fiber diets that can augment the percentage of microbes that product short chain fatty acids, ingestion of bacterial nutrients (prebiotics), and ingestion of probiotics or fecal microbial transplant that introduce “healthy” commensals (52).

#### Ulcerative colitis and Crohn’s disease

The overlapping features of ulcerative colitis/proctitis and Crohn’s disease (referred to as IBD) and CVID enteropathy make the distinctions between these diagnoses complex (53). The clinical symptoms such as weight loss, chronic diarrhea, rectal bleeding, abdominal pain, and malabsorption can be similar. On endoscopy, crypt-destructive colitis can be seen in both IBD and CVID enteropathy, and both can lead to strictures, although an underlying Crohn’s diagnosis is more likely to lead to this complication (53). On a molecular level, IL-12 and *IFN-y* can be increased in both IBD and CVID enteropathy, while CVID shows decreased IL-23 in the gut (17, 54, 55). These cytokine profiles have been linked to the presence of chronic norovirus infection and complete absence of IgA (17, 54). In our cohort, 5 subjects were diagnosed with Crohn’s disease and 7 were diagnosed with ulcerative colitis/proctitis. These patients tended to have better response to biologic therapy in comparison to those with CVID enteropathy, which also reflects previous reports (56).

#### Chronic gastritis

Several studies described the endoscopic and histologic findings of chronic gastritis in CVID patients with and without gastrointestinal symptoms, with atrophic gastritis being the most common complication, affecting ~ 30% of patients (18, 46). Interestingly, in our cohort, only 2 of 114 patients presented with chronic gastritis, which may represent eradication of *Helicobacter pylori* in the setting of repeated antibiotic use. A Finnish cohort evaluated 71 patients with CVID, of whom 15% had chronic active gastritis, and 17% had atrophic gastritis; *Helicobacter pylori* was only found in 8 (6%) patients (15). Jorgensen et al. found that antral inflammation was associated with increased sCD14 and sCD25, suggesting activation of monocytes and T-cells (18). Given these findings, it is hypothesized that other mechanisms unrelated to H. pylori may promote chronic gastric mucosal inflammation in CVID. These mechanisms could include immune dysregulation and dysbiosis, as previously mentioned (52, 57). Pernicious anemia has also been associated with CVID, affecting up to 15% of patients (58). However, parietal cell antibodies are not detected even with evidence of corpus-restricted atrophic gastritis without *H. pylori* infection, emphasizing the need for biopsy (46).

#### Nodular lymphoid hyperplasia

Histopathologic features of the bowel in CVID patients with gastrointestinal symptoms demonstrated a variety of findings, including lymphoid hyperplasia, villous atrophy, a paucity or absence of plasma cells, an increase in intraepithelial lymphocytes, or even normal mucosa (43). Nodular lymphoid hyperplasia (NLH), defined as an accumulation of multiple small nodules 1 mm or larger with a germinal center, is frequently identified on biopsies from both small and large intestines of CVID patients. In contrast, lymphocytic colitis is characterized by an increase in intraepithelial lymphocytes without germinal centers. In healthy adults, NLH is rare and usually associated with chronic infection. Although NLH is not always present, *Giardia lamblia* has been found in association with NLH in CVID patients and can be difficult to eradicate. In our cohort, although Giardia was one of the more commonly identified pathogens in chronic infection, there was no correlation between chronic Giardia infection and NLH. In a recent retrospective cohort study by van Schewick et al, a review of 95 bowel histology samples from 44 adult CVID patients were grouped by histologic patterns. In this study, three major histological patterns were observed in CVID patients with gastrointestinal symptoms - a celiac-like histology, IBD-like changes, and nodular lymphoid hyperplasia (NLH). Of all colonic biopsies, NLH was found in 47.8%, with twelve patients (34.3%) having NLH occurring alone without other histopathological features (43).

#### Genetic sequencing

With advances in genetic analyses, some of the underlying causes of CVID have now been elucidated. Across the current 728 patient cohort, a CVID related gene defect was identified in 35% of subjects, exceeding the frequency noted in several large cohorts at 25%, and 31% (59, 60). For those examined here with GI disease, 16 out of 47 who underwent whole exome sequencing demonstrated a gene defect (34%) (**Table 3**). In some cases, these findings provide clues to the pathogenesis of the complications in CVID, such as mutations in CTLA4, LRBA and BACH2 which have been identified in subjects with severe enteropathy (61, 62). While these mutations were noted in single subjects within this cohort, mutations in TACI and NFKB1 were the most common. However, it is notable that previous attempts at correlating phenotype with genotype in CVID have demonstrated significant overlap (59).

## Conclusion

Within our current CVID cohort, 114 subjects had experienced significant GI disease, including infectious and noninfectious complications. Norovirus, Giardia and CMV were the most frequently documented infections. In terms of inflammatory complications, CVID enteropathy led to chronic diarrhea and malabsorption as the main presentations. Concomitant autoimmunity, lung disease and malignancy were also present in many, underscoring the complexity of the CVID syndrome and its management.

## Data availability statement

The original contributions presented in the study are included in the article/**Supplementary Material**, further inquiries can be directed to the corresponding author/s.

## Ethics statement

The studies involving human participants were reviewed and approved by the institutional review board at the Mount Sinai Hospital. Written informed consent to participate in this study was provided to all participants.

## Author contributions

DS: Wrote the majority of the manuscript, provided the most substantial contributions to the conception, design of the work; as well as the acquisition, analysis, and interpretation of data. KR: Provided important revisions, as well as contributions to design of the work. CT: Provided important contributions to the discussion. MH: Also provided important contributions to the discussion. CC-R: As the principal investigator, she provided critical contributions to the design of the work, and acquisition of the data. She also provided the most important critical feedback upon completion of the manuscript and gave approval for publication. All authors contributed to the article and approved the submitted version.

## References

[1] Bonilla FA Barlan I Chapel H Costa-Carvalho BT Cunningham-Rundles C de la Morena MT. International consensus document (ICON): common variable immunodeficiency disorders. J Allergy Clin Immunol Pract (2016) 4:38–59. doi: 10.1016/j.jaip.2015.07.025 26563668 PMC4869529

[2] Bateman EAL Ayers L Sadler R Lucas M Roberts C Woods A. T cell phenotypes in patients with common variable immunodeficiency disorders: associations with clinical phenotypes in comparison with other groups with recurrent infections. Clin Exp Immunol (2012) 170:202–11. doi: 10.1111/j.1365-2249.2012.04643.x PMC3482367 23039891

[3] Giovannetti A Pierdominici M Mazzetta F Marziali M Renzi C Mileo AM. Unravelling the complexity of T cell abnorMalities in common variable immunodeficiency. J Immunol (2007) 178:3932–43. doi: 10.4049/jimmunol.178.6.3932 17339494

[4] Gathmann B Mahlaoui N Gérard L Oksenhendler E Warnatz K Schulze I. Clinical picture and treatment of 2212 patients with common variable immunodeficiency. J Allergy Clin Immunol (2014) 134:116–26. doi: 10.1016/j.jaci.2013.12.1077 24582312

[5] Resnick ES Moshier EL Godbold JH Cunningham-Rundles C. Morbidity and mortality in common variable immune deficiency over 4 decades. Blood (2012) 119:1650–7. doi: 10.1182/blood-2011-09-377945 PMC3286343 22180439

[6] Ameratunga R Gillis D Steele R. Diagnostic criteria for common variable immunodeficiency disorders. J Allergy Clin Immunol Pract (2016) 4:1017–8. doi: 10.1016/j.jaip.2016.02.023 27587325

[7] Baloh C Reddy A Henson M Prince K Buckley R Lugar P. 30-year review of pediatric- and adult-onset CVID: clinical correlates and prognostic indicators. J Clin Immunol (2019) 39:678–87. doi: 10.1007/s10875-019-00674-9 PMC6754754 31377970

[8] Chapel H Lucas M Lee M Bjorkander J Webster D Grimbacher B. Common variable immunodeficiency disorders: division into distinct clinical phenotypes. Blood (2008) 112:277–86. doi: 10.1182/blood-2007-11-124545 18319398

[9] Gonzalez-Quintela A Alende R Gude F Campos J Rey J Meijide LM. Serum levels of immunoglobulins (IgG, IgA, IgM) in a general adult population and their relationship with alcohol consumption, smoking and common metabolic abnorMalities. Clin Exp Immunol (2008) 151:42–50. doi: 10.1111/j.1365-2249.2007.03545.x 18005364 PMC2276914

[10] Ahn S Cunningham-Rundles C. Role of B cells in common variable immune deficiency. Expert Rev Clin Immunol (2009) 5:557–64. doi: 10.1586/eci.09.43 PMC2922984 20477641

[11] Sánchez-Ramón S Radigan L Yu JE Bard S Cunningham-Rundles C. Memory B cells in common variable immunodeficiency: clinical associations and sex differences. Clin Immunol (2008) 128:314–21. doi: 10.1016/j.clim.2008.02.013 PMC2692232 18620909

[12] Warnatz K Denz A Dräger R Braun M Groth C Wolff-Vorbeck G. Severe deficiency of switched memory B cells (CD27(+)IgM(-)IgD(-)) in subgroups of patients with common variable immunodeficiency: a new approach to classify a heterogeneous disease. Blood (2002) 99:1544–51. doi: 10.1182/blood.V99.5.1544 11861266

[13] Wehr C Kivioja T Schmitt C Ferry B Witte T Eren E. The EUROclass trial: defining subgroups in common variable immunodeficiency. Blood (2008) 111:77–85. doi: 10.1182/blood-2007-06-091744 17898316

[14] Reichert T DeBruyère M Deneys V Tötterman T Lydyard P Yuksel F. Lymphocyte subset reference ranges in adult Caucasians. Clin Immunol Immunopathol (1991) 60:190–208. doi: 10.1016/0090-1229(91)90063-G 1712687

[15] Pikkarainen S Martelius T Ristimäki A Siitonen S Seppänen MRJ Färkkilä M. A high prevalence of gastrointestinal manifestations in common variable immunodeficiency. Am J Gastroenterol (2019) 114:648–55. doi: 10.14309/ajg.0000000000000140 PMC6455088 30747770

[16] Barmettler S Ong M-S Farmer JR Yang N Cobbold M Walter JE. Gastrointestinal manifestations in common variable immunodeficiency (CVID) are associated with an altered immunophenotype including B- and T-cell dysregulation. J Allergy Clin Immunol Pract (2020) 8:1436–1438.e1. doi: 10.1016/j.jaip.2019.10.024 31704440 PMC7438162

[17] Shulzhenko N Dong X Vyshenska D Greer RL Gurung M Vasquez-Perez S. CVID enteropathy is characterized by exceeding low mucosal IgA levels and interferon-driven inflammation possibly related to the presence of a pathobiont. Clin Immunol (2018) 197:139–53. doi: 10.1016/j.clim.2018.09.008 PMC6289276 30240602

[18] Malamut G Verkarre V Suarez F Viallard J-F Lascaux A-S Cosnes J. The enteropathy associated with common variable immunodeficiency: the delineated frontiers with celiac disease. Am J Gastroenterol (2010) 105:2262–75. doi: 10.1038/ajg.2010.214 20551941

[19] Maarschalk-Ellerbroek LJ Oldenburg B Mombers IMH Hoepelman AIM Brosens LAA Offerhaus GJA. Outcome of screening endoscopy in common variable immunodeficiency disorder and X-linked agammaglobulinemia. Endoscopy (2013) 45:320–3. doi: 10.1055/s-0032-1326078 23325698

[20] Mormile I Punziano A Riolo CA Granata F Williams M de Paulis A. Common variable immunodeficiency and autoimmune diseases: A retrospective study of 95 adult patients in a single tertiary care center. Front Immunol (2021) 12:652487. doi: 10.3389/fimmu.2021.652487 34290696 PMC8287325

[21] Pecoraro A Crescenzi L Varricchi G Marone G Spadaro G. Heterogeneity of liver disease in common variable immunodeficiency disorders. Front Immunol (2020) 11:338. doi: 10.3389/fimmu.2020.00338 32184784 PMC7059194

[22] Song J Crescenzi L Varricchi G Marone G Spadaro G. Common variable immunodeficiency and liver involvement. Clin Rev Allergy Immunol (2018) 55:340–51. doi: 10.1007/s12016-017-8638-z PMC5803456 28785926

[23] Ardeniz O Cunningham-Rundles C. Granulomatous disease in common variable immunodeficiency. Clin Immunol (2009) 133:198–207. doi: 10.1016/j.clim.2009.05.001 19716342 PMC2760682

[24] Bates CA Lleo A Yang GX Zhang W Bowlus CL Gershwin ME. Granulomatous-lymphocytic lung disease shortens survival in common variable immunodeficiency. J Allergy Clin Immunol (2004) 114:415–21. doi: 10.1016/j.jaci.2004.05.057 15316526

[25] Agarwal S Cunningham-Rundles C. Autoimmunity in common variable immunodeficiency. Ann Allergy Asthma Immunol (2019) 123:454–60. doi: 10.1016/j.anai.2019.07.014 PMC7310570 31349011

[26] Cunningham-Rundles C. Common variable immunodeficiency. Curr Allergy Asthma Rep (2001) 1:421–9. doi: 10.1007/s11882-001-0027-1 11892068

[27] Kiaee F Azizi G Rafiemanesh H Zainaldain H Sadaat Rizvi F Alizadeh M. Malignancy in common variable immunodeficiency: a systematic review and meta-analysis. Expert Rev Clin Immunol (2019) 15:1105–13. doi: 10.1080/1744666X.2019.1658523 31452405

[28] Brown L-AK Clark I Brown JR Breuer J Lowe DM. Norovirus infection in primary immune deficiency. Rev Med Virol (2017) 27:e1926. doi: 10.1002/rmv.1926 28271593

[29] Woodward J Gkrania-Klotsas E Kumararatne D. Chronic norovirus infection and common variable immunodeficiency. Clin Exp Immunol (2017) 188:363–70. doi: 10.1111/cei.12884 PMC5422859 27753065

[30] Russmann S Grattagliano I Portincasa P Palmieri VO Palasciano G. Ribavirin-induced anemia: mechanisms, risk factors and related targets for future research. Curr Med Chem (2006) 13:3351–7. doi: 10.2174/092986706778773059 17168855

[31] Brown L-AK Ruis C Clark I Roy S Brown JR Albuquerque AS. A comprehensive characterization of chronic norovirus infection in immunodeficient hosts. J Allergy Clin Immunol (2019) 144:1450–3. doi: 10.1016/j.jaci.2019.07.036 PMC6843911 31415785

[32] Díaz-Alberola I Gutiérrez-Bautista JF Espuch-Oliver A García-Aznar JM Anderson P Jiménez P. Incidence, management experience and characteristics of patients with giardiasis and common variable immunodeficiency. J Clin Med (2022) 11. doi: 10.3390/jcm11237007 PMC9740678 36498582

[33] Barsotti NS Almeida RR Costa PR Barros MT Kalil J Kokron CM. IL-10-producing regulatory B cells are decreased in patients with common variable immunodeficiency. PloS One (2016) 11:e0151761. doi: 10.1371/journal.pone.0151761 26991898 PMC4798727

[34] Dann SM Le CHY Hanson EM Ross MC Eckmann L. Giardia infection of the small intestine induces chronic colitis in genetically susceptible hosts. J Immunol (2018) 201:548–59. doi: 10.4049/jimmunol.1700824 PMC7351291 29898958

[35] Oksenhendler E Gérard L Fieschi C Malphettes M Mouillot G Jaussaud R. Infections in 252 patients with common variable immunodeficiency. Clin Infect Dis (2008) 46:1547–54. doi: 10.1086/587669 18419489

[36] Bourque DL Neumayr A Libman M Chen LH. Treatment strategies for nitroimidazole-refractory giardiasis: a systematic review. J Travel Med (2022) 29:taab120. doi: 10.1093/jtm/taab120 34350966

[37] Cannon MJ Schmid DS Hyde TB. Review of cytomegalovirus seroprevalence and demographic characteristics associated with infection. Rev Med Virol (2010) 20:202–13. doi: 10.1002/rmv.655 20564615

[38] Godsell J Chan S Slade C Bryant V Douglass JA Sasadeusz J. Cytomegalovirus in primary immunodeficiency. Curr Opin Infect Dis (2021) 34:663–71. doi: 10.1097/QCO.0000000000000797 34608876

[39] Chan S Godsell J Horton M Farchione A Howson LJ Margetts M. Case report: cytomegalovirus disease is an under-recognized contributor to morbidity and mortality in common variable immunodeficiency. Front Immunol (2022) 13:815193. doi: 10.3389/fimmu.2022.815193 35242131 PMC8885594

[40] Bright PD Gompels M Donati M Johnston S. Successful oral treatment of Ganciclovir resistant cytomegalovirus with Maribavir in the context of primary immunodeficiency: First case report and review. J Clin Virol (2017) 87:12–6. doi: 10.1016/j.jcv.2016.12.006 27987421

[41] Yazdani R Habibi S Sharifi L Azizi G Abolhassani H Olbrich P. Common variable immunodeficiency: epidemiology, pathogenesis, clinical manifestations, diagnosis, classification, and management. J Investig Allergol Clin Immunol (2020) 30:14–34. doi: 10.18176/jiaci.0388 30741636

[42] Dion J Malphettes M Bénéjat L Mégraud F Wargnier A Boutboul D. Campylobacter infection in adult patients with primary antibody deficiency. J Allergy Clin Immunol Pract (2019) 7:1038–1041.e4. 29981862 10.1016/j.jaip.2018.06.014

[43] van Schewick CM Lowe DM Burns SO Workman S Symes A Guzman D. Bowel histology of CVID patients reveals distinct patterns of mucosal inflammation. J Clin Immunol (2022) 42:46–59. doi: 10.1007/s10875-021-01104-5 34599484 PMC8821476

[44] Ho H-E Cunningham-Rundles C. Seeking relevant biomarkers in common variable immunodeficiency. Front Immunol (2022) 13:857050. doi: 10.3389/fimmu.2022.857050 35359997 PMC8962738

[45] Ho H-E Radigan L Bongers G El-Shamy A Cunningham-Rundles C. Circulating bioactive bacterial DNA is associated with immune activation and complications in common variable immunodeficiency. JCI Insight (2021) 6. doi: 10.1172/jci.insight.144777 PMC8525635 34622805

[46] Jørgensen SF Reims HM Frydenlund D Holm K Paulsen V Michelsen AE. A cross-sectional study of the prevalence of gastrointestinal symptoms and pathology in patients with common variable immunodeficiency. Am J Gastroenterol (2016) 111:1467–75. doi: 10.1038/ajg.2016.329 27527747

[47] Biagi F Bianchi I Zilli A Marchese A Luinetti O Lougaris V. The significance of duodenal mucosal atrophy in patients with common variable immunodeficiency: a clinical and histopathologic study. Am J Clin Pathol (2012) 138:185–9. doi: 10.1309/AJCPEIILH2C0WFYE 22904128

[48] Venhoff N Emmerich F Neagu M Salzer U Koehn C Driever S. The role of HLA DQ2 and DQ8 in dissecting celiac-like disease in common variable immunodeficiency. J Clin Immunol (2013) 33:909–16. doi: 10.1007/s10875-013-9892-3 23609110

[49] Boland BS Riedl MA Valasek MA Crowe SE Sandborn WJ. Vedolizumab in patients with common variable immune deficiency and gut inflammation. Am J Gastroenterol (2017) 112:1621. doi: 10.1038/ajg.2017.246 PMC6389596 28978958

[50] Sifers T Hirten R Mehandru S Ko HM Colombel J-F Cunningham-Rundles C. Vedolizumab therapy in common variable immune deficiency associated enteropathy: A case series. Clin Immunol (2020) 212:108362. doi: 10.1016/j.clim.2020.108362 32058070 PMC7310569

[51] Chua I Standish R Lear S Harbord M Eren E Raeiszadeh M. Anti-tumour necrosis factor-alpha therapy for severe enteropathy in patients with common variable immunodeficiency (CVID). Clin Exp Immunol (2007) 150:306–11. doi: 10.1111/j.1365-2249.2007.03481.x PMC2219360 17822445

[52] Poto R Laniro G de Paulis A Spadaro G Marone G Gasbarrini A. Is there a role for microbiome-based approach in common variable immunodeficiency? Clin Exp Med 1–18 (2023). doi: 10.1007/s10238-023-01006-3 PMC9897624 36737487

[53] Malesza IJ Malesza M Krela-Kaźmierczak I Zielińska A Souto EB Dobrowolska A. Primary humoral immune deficiencies: overlooked mimickers of chronic immune-mediated gastrointestinal diseases in adults. Int J Mol Sci (2020) 21. doi: 10.3390/ijms21155223 PMC7432083 32718006

[54] Strohmeier V Andrieux G Unger S Pascual-Reguant A Klocperk A Seidl M. Interferon-driven immune dysregulation in common variable immunodeficiency-associated villous atrophy and norovirus infection. J Clin Immunol (2023) 43:371–90. doi: 10.1007/s10875-022-01379-2 PMC9892141 36282455

[55] Mannon PJ Fuss IJ Dill S Friend J Groden C Hornung R. Excess IL-12 but not IL-23 accompanies the inflammatory bowel disease associated with common variable immunodeficiency. Gastroenterology (2006) 131:748–56. doi: 10.1053/j.gastro.2006.06.022 16952544

[56] Alventosa Mateu C Ruiz Sánchez L Amorós García C. Ulcerative colitis in?a?patient with common variable immunodeficiency: does the treatment differ from the routine? Rev Esp Enferm Dig (2016) 108:235–6. doi: 10.17235/reed.2016.4115/2015 26912253

[57] Berbers R-M Nierkens S van Laar JM Bogaert D Leavis HL. Microbial dysbiosis in common variable immune deficiencies: evidence, causes, and consequences. Trends Immunol (2017) 38:206–16. doi: 10.1016/j.it.2016.11.008 28017520

[58] Zullo A Romiti A Rinaldi V Vecchione A Tomao S Aiuti F. Gastric pathology in patients with common variable immunodeficiency. Gut (1999) 45:77–81. doi: 10.1136/gut.45.1.77 10369708 PMC1727591

[59] Abolhassani H Hammarström L Cunningham-Rundles C. Current genetic landscape in common variable immune deficiency. Blood (2020) 135:656–67. doi: 10.1182/blood.2019000929 PMC7046605 31942606

[60] Rojas-Restrepo J Caballero-Oteyza A Huebscher K Haberstroh H Fliegauf M Keller B. Establishing the molecular diagnoses in a cohort of 291 patients with predominantly antibody deficiency by targeted next-generation sequencing: experience from a monocentric study. Front Immunol (2021) 12:786516. doi: 10.3389/fimmu.2021.786516 34975878 PMC8718408

[61] ThaventhIran JED Lango Allen H Burren OS Rae W Greene D Staples E. Whole-genome sequencing of a sporadic primary immunodeficiency cohort. Nature (2020) 583:90–5. doi: 10.1038/s41586-020-2265-1 PMC7334047 32499645

[62] Afzali B Grönholm J Vandrovcova J O’Brien C Sun H-W Vanderleyden I. BACH2 immunodeficiency illustrates an association between super-enhancers and haploinsufficiency. Nat Immunol (2017) 18:813–23. doi: 10.1038/ni.3753 PMC5593426 28530713

